# Surface processes forcing on extensional rock melting

**DOI:** 10.1038/s41598-020-63920-w

**Published:** 2020-05-07

**Authors:** Pietro Sternai

**Affiliations:** Department of Earth and Environmental Sciences, University of Milano-Bicocca, Milan, Italy

**Keywords:** Geodynamics, Geology, Geomorphology, Tectonics

## Abstract

Surface processes and magmatism condition the structural evolution of continental rifts and passive margins through mechanical and thermal effects on the lithosphere rheology. However, their inter-relationships in extensional settings are largely unknown. Here, I use coupled thermo-mechanical geodynamic and landscape evolution numerical modeling to assess the links between erosion of rift shoulders, sedimentation within the rift basin and extensional rock melting. Results suggest that, when the crust is thinner than ~40 km, the extension rate is slower than ~2 cm/yr and the mantle potential temperature is below ~1230 °C, efficient surface processes may double crustal melting by Moho lowering and inhibit mantle decompression melting by ~50% through sediment loading within the rift basin. It is thus likely that surface processes significantly influenced the magmatic activity of a number of extensional settings worldwide – e.g. the Mediterranean, the Gulf of California, the Iberia-Newfoundland margin, and the South China Sea. Because magmatism and surface processes affect jointly the geological carbon cycle, the surface processes forcing on extensional rock melting investigated here involves an additional means of linkage between plate tectonics and climate changes.

## Introduction

**Figure 1:**
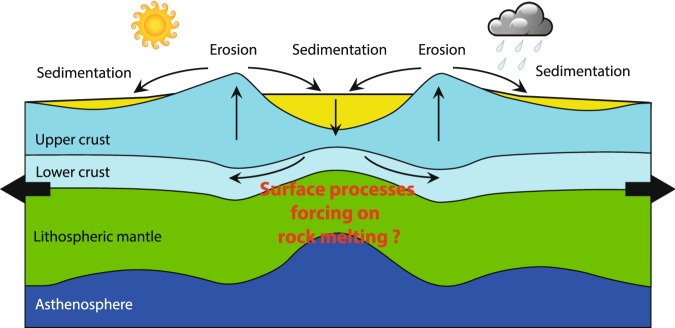
Problem setting: Schematic representation of the interrelationship between surface processes and lithospheric strain in an extensional setting (not to scale, modified after ref. ^4^). Sediments derived from erosion of rift shoulders load the rift basins. The rigid upper crust and lithospheric mantle lithosphere flex and weaken, while more ductile lower-crustal material flows from the centre of the rift outward, facilitating uplift and erosion of the rift shoulders. The associated effects on extensional rock melting are to date poorly constrained. The figure was generated with Adobe Illustrator version 24.0.1.

Continental extension, rifting and breakup involve the formation of thick sedimentary basins and escarpments at rift flanks that rise even a few kilometres above sea level. Progressive thinning and cooling of a heated lithosphere, strain localization, small-scale mantle convection and flexural forces are classically considered as some of the most important tectonic processes controlling the evolution of these prominent topographic features^1–3^. In turn, acting at similar rates to those of tectonic processes and generating stresses in the order of hundreds of MPa, sedimentation into the rift basin of the material eroded from the uplifted flanks affects the rheological response of the lithosphere to the tectonic forcing^4–7^. Enhanced basin subsidence by outward flow of the lower viscous crust due to sediment loading and enhanced uplift of the rift shoulders by surface unloading due to erosion are amongst the most relevant rheological implications of surface processes during rifting^4^ (Fig. 1).

Magmatism is a distinctive feature of continental lithospheric stretching^8,9^. Depending on the volumes of magma produced, two end-member types of rifted margins can be defined: volcanic or magma-poor^10^. At volcanic rifted margins, voluminous magmas erupt or intrude crustal units over short times, usually during continental breakup^11,12^, although the mechanisms responsible for the production of large volumes of magmas and the active contribution of mantle plumes to rifting are controversial^13,14^. Magma-poor rifted margins are characterized by wide domains of extended lithosphere with local magmatic bodies intruding syn-rift and especially post-breakup units^10,15,16^. In both cases, because the stresses required to rupture a typical continental lithosphere are estimated to be higher than those generated by extensional tectonics^17,18^, lithospheric heating/weakening and magma production likely provide a substantial contribution to lithospheric rupturing^19–21^. For instance, the magma migration path is expected to follow fractures along forming fault zones^22,23^, and fluid supply will then increase the pore fluid pressure, thereby lowering the plastic yield strength of fractured rocks and further localising the strain along weakening fault zones^24–26^.

While the relationships between lithospheric strain and surface processes and between lithospheric strain and magmatism are classically the subject of intense research^4,27–32^, the links between surface processes and rock melting across extensional settings are poorly constrained (Fig. 1). Yet, peaks of igneous activity due to enhanced mantle decompression melting have been ascribed to surface unloading by ongoing deglaciation^33–35^ and associated erosion^36^ or sea level lowering^37,38^. Modifications of the surface topography during the structural evolution of a magmatic province may affect the stress pattern within the elastic upper crust, with associated effects on the propagation of dykes^39^, the exsolution of magmatic gasses, and the pressure/overpressure field^40^. These factors co-determine the probability of magmatic, volcanic, and degassing events to occur as well as the amount of volatiles released into the ocean and atmosphere^41^. Because carbon degassing from extensional settings influenced climate changes at geological timescales^42,43^ and climate exerts a primary control on surface processes^27^, assessing likely relationships between surface processes and rock melting in rifting contexts is timely and key to our understanding of the geological carbon cycle and the surface-deep Earth processes coupling^44^.

In this study, I use coupled thermo-mechanical and landscape evolution numerical modeling to investigate the role of erosion and sedimentation in affecting the magmatic activity during continental extension and rifting. Given a set of experimentally/petrologically-determined rheological and partial rock melting relationships and parameters, one may simulate a range of plausible continental rift histories accounting for variable prescribed extension rates, crustal thickness, mantle potential temperature (*sensu* ref. ^45^) and erosion/sedimentation rates. The comparison between models enables assessing under which conditions surface processes can affect extensional rock melting as well as the mechanisms and feedbacks involved.

### The numerical model

The main question addressed here is: can the surface mass redistribution by erosion and sedimentation affect partial melting of a stretching continental lithosphere? The general model includes three main components: (1) a surface process model, (2) a rheological lithospheric model, and (3) a model for partial rock melting. The evolution of the surface loads due to erosion and sedimentation is based on empirically determined laws of the surface transport of geological materials^46,47^. Rheological and partial melting laws/parameters are based on experimental rock mechanics and petrology^48,49^. The different components of the model are described in more detail hereafter.

### Surface processes

**Figure 2:**
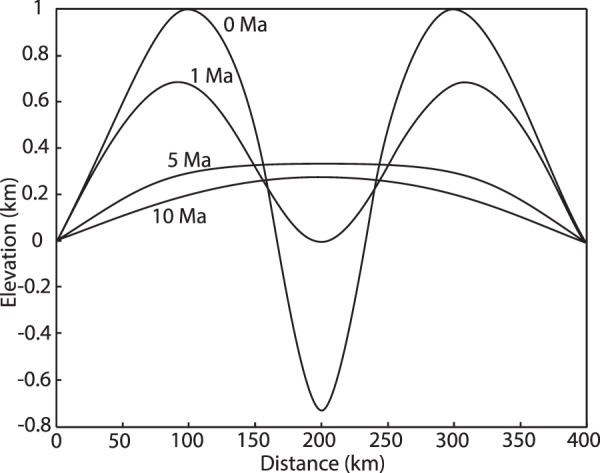
Evolution of a synthetic topography across a stretching lithosphere: Continental rift shoulders and basin evolution under the assumption that the landscape is controlled by a linear diffusion equation (Eq. 1). The figure was generated with MATLAB version R2017 and assembled with Adobe Illustrator version 24.0.1.

The simplest representation of surface processes consists in the removal of rocks from uplifted regions (erosion), transport of the eroded material to the nearby basins and deposition within them (sedimentation). When integrated over long timescales (>10^5^ yr) and large length scales (>10^5^ m), these processes can be jointly reproduced by a linear downslope diffusion equation applied to the evolving topography in response to the tectonic strain^4,7,47,50^. The sediment flux at the surface, ${{\rm{q}}}_{s}$, is related to the local slope, $\nabla z$, by ${{\rm{q}}}_{s}=k\nabla z$. The effective diffusivity, $k$, given by the velocity of transport of the eroded material multiplied by its thickness, is a measure of the efficiency of surface processes. The assumption of mass conservation leads to the linear diffusion equation for erosion and sedimentation,

1 $$\partial z/\partial t=k{\nabla }^{2}z$$

where $t$ is time. Equation 1 can be solved numerically through integration on a discrete topography with constant-elevation boundary conditions^4,50^ (Fig. 2), thereby simulating the smoothing of the landscape by the cumulative effect of erosion and sediment deposition^46,47^. At each time step, the modelled landscape is updated for the effects of erosion/sedimentation and the associated surface load changes are computed.

### Rheology

This model component accounts for the visco-elasto-plastic rheology of the lithosphere based on rock mechanics data for a quartz-dominated crust and an olivine-dominated mantle^48^. This implies a power law stress and exponential temperature dependence of the strain rate within the ductile part of the lithosphere. The elastic behaviour is given by linearly related stress and strain assuming typical Young’s modulus, $\mu$, and Poisson’s ratio, $\nu$, while a linear dependence between the brittle rock strength and pressure is assumed for plastic deformation (Table 1).

**Table 1:** Material properties used in all numerical experiments. ρ_0_ (of solid and molten material) is the density, E_a_ is the activation energy, V_a_ is the activation volume, *n* is the stress exponent, C is the cohesion, Qz. and Ol. correspond to the abbreviations of Quartzite and Olivine, φ_eff_ is the effective internal friction angle, c is the thermal conductivity, $\mu$ is the Young’s modulus, $\nu$ is the Poisson’s ratio, C_p_ is the specific heat capacity, H_r_ is the radiogenic heat production, $\alpha$ and $\beta$ are the thermal expansion and compressibility, respectively, ${Q}_{l}$ is the lithology-related latent heating, and ${T}_{s}$ and ${T}_{l}$ are the solidus and liquidus temperature, respectively. Values are taken from refs. ^51,55^ and references therein.

	${{\boldsymbol{\rho }}}_{0}^{{\boldsymbol{s}}}\,{{\boldsymbol{\rho }}}_{0}^{{\boldsymbol{l}}}\,$ (kg/m^3^)	Ea (kJ/mol)	Va (m^3^/mol)	n	C (MPa)	Viscous flow law	sin(ϕ_eff_)	c (W/m/K)	μ (GPa)	v	C_p_ (J/kg/K)	H_r_ (μW/m^3^)	α(1/K)	β(1/Pa)	Q_l_ (kJ/kg)	T_solidus_ (K)	T_liquidus_ (K)
Sediments	2700 (solid), 2400 (molten)	154	8	2.3	10	Wet Qz.	0.15	0.64 + 807/(T + 77)	10	0.2	1000	1	3x10^–5^	1x10^–11^	300	889 + 17900/(P + 54)+20200/(P + 54)2 at P < 1200 MPa, 831 + 0.06 P at P > 1200 MPa	1262 + 0.09 P
Continental crust	2800 (solid), 2400 (molten)	154	8	2.3	10	Wet Qz.	0.2	0.64 + 807/(T + 77)	10	0.2	1000	1	3x10^–5^	1x10^–11^	300	889 + 17900/(P + 54)+20200/(P + 54)2 at P < 1200 MPa, 831 + 0.06 P at P > 1200 MPa	1262 + 0.09 P
Mantle	3300 (solid), 2700 (molten)	532	8	3.5	10	Dry Ol.	0.6	0.73 + 1293/(T + 77)	67	0.2	1000	0.02	3x10^–5^	1x10^–11^	400	1394 + 0.132899P-0.000005104P2 at P < 1000 MPa, 2212 + 0.030819(P-10000) at P > 1200 MPa	2073 + 0.114 P

The structural changes of an evolving lithosphere are governed by the mass, momentum and energy conservation equations,

2 $$\{\begin{array}{c}\partial \rho /\partial t+div(\partial v)=0\\ div(\sigma )+\rho g=0\\ \rho {C}_{p}\cdot \partial T/\partial t-div(c\nabla T)+v\nabla T={H}_{r}+{H}_{s}+{H}_{a}+\,{H}_{l}\end{array}$$

where $\rho$ is the density, ${\boldsymbol{v}}$ is the velocity tensor, ${\boldsymbol{\sigma }}$ is the stress tensor, $g$ is the acceleration due to gravity, ${C}_{p}$ is the specific heat capacity, $T$ is temperature, $c$ is the thermal conductivity, and ${H}_{r}$, ${H}_{s}$, ${H}_{a}$, and ${H}_{l}$ are the radiogenic, shear, adiabatic and latent heat production per unit volume, respectively^51^. Values for ${H}_{r}$ are listed in Table 1. ${H}_{s}$, related to dissipation of the mechanical energy during irreversible deformation, is calculated as ${H}_{s}={\sigma }_{ij}^{\text{'}}\dot{{\varepsilon }_{ij}^{\text{'}}}$, where ${\boldsymbol{\sigma }}{\boldsymbol{{\prime} }}$ is the deviatoric stress tensor, $\dot{{\boldsymbol{\varepsilon }}{\boldsymbol{{\prime} }}}$ is the deviatoric strain rate tensor, *i* and *j* are coordinate indices (x, y) and repeated *ij* indices denotes summation. The adiabatic heat production, related to changes in pressure, is calculated as ${H}_{a}=T\alpha \frac{DP}{Dt}$, where $\,\alpha$ is the thermal expansion. ${H}_{l}$, due to rock melting and crystallisation, is accounted for by computing the effective heat capacity, $C{p}_{eff}$, and thermal expansion, ${\alpha }_{eff}$, of partially molten rocks (0 < $\xi$ ≤ 1, see following) as^52^:

3 $$C{p}_{eff}=Cp+{Q}_{l}{\left(\frac{\partial \xi }{\partial T}\right)}_{P=const}$$

4 $${\alpha }_{eff}=\alpha +\frac{{Q}_{l}}{T}{\rho }_{eff}{\left(\frac{\partial \xi }{\partial P}\right)}_{T=const}$$

where ${Q}_{l}$ is the lithology-related latent heating (Table 1) and $\xi$ is the volumetric fraction of melt computed as described hereafter.

### Partial rock melting

The numerical model allows for partial melting and crystallisation of magma in the pressure-temperature domain between the wet solidus and dry liquidus of corresponding rocks (Table 1)^49,53,54^. At constant pressure, $\xi$ is assumed to increase linearly with temperature according to the relations^52^.

5 $$\{\begin{array}{c}\xi =0\,at\,T\,\le {T}_{s}\\ \xi =\frac{(T-{T}_{s})}{({T}_{l}-{T}_{s})}\,at\,{T}_{s} < T < {T}_{l}\\ \xi =1\,at\,T\ge {T}_{l}\end{array}$$

where ${T}_{s}$ and ${T}_{l}$ are the solidus and liquidus of the considered rock, respectively. The effective density, ${\rho }_{eff}$, of partially molten rocks is then calculated as

6 $${\rho }_{eff}={\rho }_{s}\,\left(1-\xi +\xi \frac{{\rho }_{l}^{0}}{{\rho }_{s}^{0}}\right)$$

where ${\rho }_{s}^{0}$ and ${\rho }_{l}^{0}$ are the standard densities of solid and molten rocks, respectively, and ${\rho }_{s}={\rho }_{0}[1+\beta (P-{P}_{0})]\times [1-\alpha (T-{T}_{0})]$ is the density of solid rocks at given P-T conditions (where $\beta$ is the compressibility, P is pressure, and ${\rho }_{0}$, ${P}_{0}$ and ${T}_{0}$ are the density, pressure and temperature of rocks at surface conditions) (Table 1). The effective viscosity, $\eta$, of partially molten rocks with $\xi$ > 0.1 is assigned a low constant value of 10^16^ Pa s, which is an oversimplification, but it allows obviating numerical hurdles due to too high viscosity jumps between different materials^55^.

### Integrated model components, reference setup and boundary conditions

**Figure 3:**
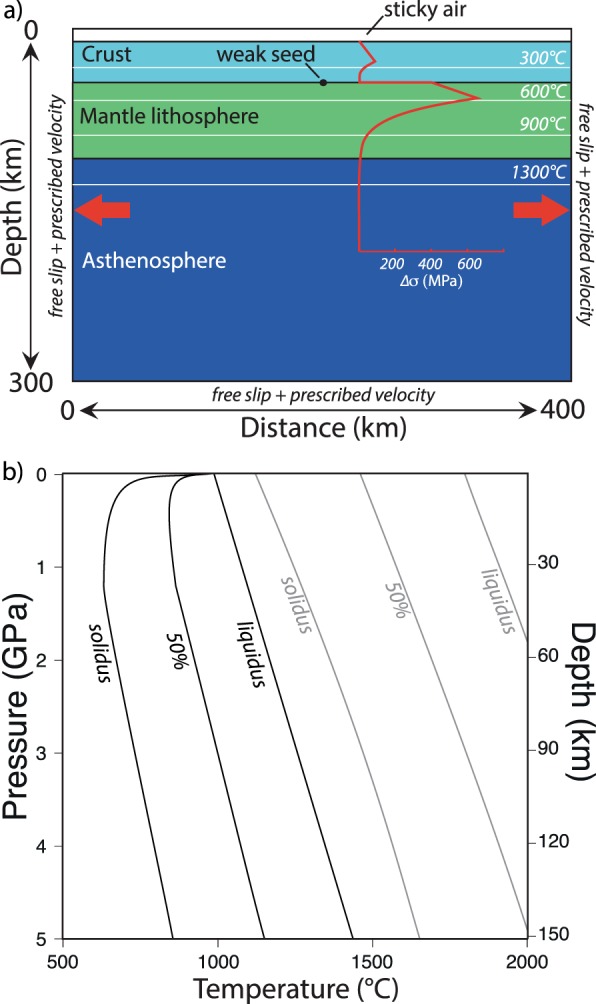
Model setup: (**a**) Initial model domain, boundary conditions and yield strength profile. Colours show different rock types. (**b**) Assumed solidus and liquidus for crustal (black)^53,54^ and mantle (grey)^49^ material. See also ref. ^55^. and references therein, Table 1, Table 2 and text for details. The figure was generated with MATLAB version R2017 and assembled with Adobe Illustrator version 24.0.1.

Equations 1, 2 and 5 are coupled via stress, temperature and velocity continuity conditions, adopting the finite-differences approximation scheme and a fully staggered bi-dimensional grid, following the approach described in refs. ^25^ and^55^. The initial domain (Fig. 3) measures 400 × 300 km in the x and y dimensions, resolved by 161 × 61 grid points respectively, distributed on an irregular Eulerian grid that accounts for a resolution of 2 km along both directions in the central-upper part of the model. 400x300 Lagrangian markers are randomly distributed in the x and y dimensions and used for advecting the material properties. The material properties carried by Lagrangian markers are then interpolated onto the Eulerian grid via a 4^th^ order Runge-Kutta interpolation scheme. The reference model includes a 100 km thick continental lithosphere and a 35 km thick continental crust, consistently with Moho depths commonly observed at continental margins in global crustal models^56^. The velocity boundary conditions are free slip at all boundaries (x = 0 and x = 400 km; y = 0 and y = 300 km). The left and right boundaries (x = 0 and x = 400 km) also account for x-parallel velocities, which define the extension rate within the model being equally distributed on the two boundaries so that the total extension rate measures up to a few cm/yr (Table 2), in agreement with common plate velocity values^57^. The lower boundary (y = 300 km) also accounts for y-parallel velocity to compensate for horizontal extension and ensure global mass conservation. The top surface of the lithosphere is calculated dynamically as an internal free surface through a 10 km thick layer of “sticky air”^55^. The initial temperature gradient in the asthenospheric mantle is 0.4 °C/km (adiabatic)^51^. The thermal boundary conditions are 0 °C for the upper boundary, nil horizontal heat flux across the vertical boundaries, and temperatures between 1320–1420 °C at the lower model boundary in order to account for different mantle potential temperatures (see Table 2 and a discussion about plausible mantle potential temperature in section 4). Except for the initial thermal state, there are no temperature conditions imposed within the model domain throughout the simulations. A seed of week material with 2 km radius is imposed at the centre of the model domain at the Moho to initiate the lithospheric rupture. At each time step, which is limited by the Courant criteria^58^, vertical topographic load changes are computed based on Eq. 1.

**Table 2:** Parametric study summary. 36 numerical experiments with different combinations of initial Moho depth, y_moho_, mantle potential temperature, T_pot_, extensional velocities, V_ext_, and effective diffusivity, *k*, were performed and analysed. Values are based on refs. ^4,55–57^ and references therein.

	y_moho_ (km)	T_pot_ (°C)	V_ext_ (cm/yr)	*k* (mm/yr)
RUN 1 (ref)	35	1200	1	0.1–10
RUNS 2–36	35–40–45	1200–1250–1300	1–2–3	0.1–10

The parametric study focuses on the imposed extensional velocity, crustal thickness and mantle potential temperature (Table 2), which are arguably amongst the most important parameters controlling the structural and magmatic evolution of continental rifts^4,20,21^. Note that, because compositional changes between the lithospheric and asthenospheric mantle are not accounted for (Table 1), the parametric study on the mantle potential temperature also provides insights about the effects of changing the lithospheric thickness, inherently set by the depth of the 1300 °C isotherm^59^. For each combination of imposed conditions, one simulation with efficient and one with inefficient surface processes (controlled by the effective diffusivity, $k$, in Eq. 1) are performed. A total of 36 numerical experiments were performed and analysed, results are summarized in the following.

## Results

**Figure 4:**
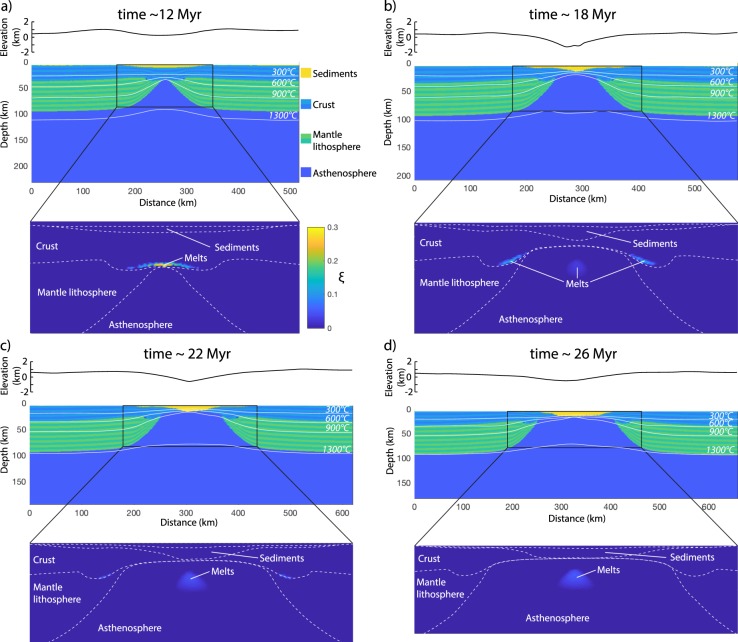
Evolution of the reference model: Selected snapshot of the reference model evolution at ~12 (**a**), 18 (**b**), 22 (**c**) and 26 (**d**) Myr. Top, middle and lower panel show the surface topography, lithology distribution and amount of molten material, respectively. In the middle panel, the light-dark colour layering in the crust and mantle lithosphere facilitates the strain visualization. The inset on the middle panel shows the location of the lower panel. The figure was generated with MATLAB version R2017 and assembled with Adobe Illustrator version 24.0.1.

**Figure 5:**
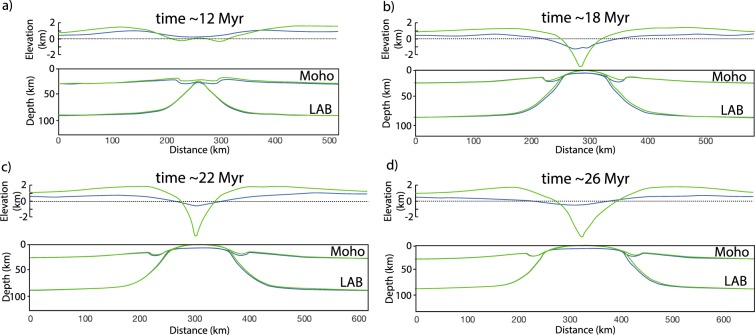
Topography, Moho and lithosphere-asthenosphere boundary (LAB) evolution: Comparison between the topography, Moho and LAB at ~12 (**a**), 18 (**b**), 22 (**c**) and 26 (**d**) Myr of the reference model when efficient ($k=10$, blue lines) and inefficient ($k=0.1$, green lines) surface processes are accounted for. Note that sediments within the rift basin when efficient surface processes are accounted for ($k=10$, blue lines) prevent the Moho to reach the surface even during advance stages of the simulation. The figure was generated with MATLAB version R2017 and assembled with Adobe Illustrator version 24.0.1.

The evolution of the reference model is shown in Fig. 4. The details and timing of the model evolution depend upon the assumed thermal and mechanical parameters (see following), but the overall sequence of events, coherent with that of “type II” continental rifts following the classification of ref. ^60^ is robust and includes: (1) lithospheric stretching, early breakup of the mantle lithosphere and asthenospheric upwelling to Moho depths, (2) prolonged crustal stretching to continental breakup and oceanization between the two newly formed continental passive margins. Faster lithospheric mantle than crustal thinning leads to heating of the Moho and the production of migmatitic core complexes and/or andesitic/dacitic effusive rocks by basal crustal melting in the early stages of the model evolution. Similar early extensional products were modeled before^61^ and observed, for instance, in the Rhodopes, the Vøring Plateau and the Basin and Range^62–65^. Crustal melting in latter two cases is driven by heating from basaltic intrusions due to active upwelling of hot asthenosphere^63,65^. Here, crustal melting rather occurs by syn-extensional diffusive heating from the asthenosphere, more similar to, for instance, the Eocene Rhodope migmatitic core complexes^62^. Regardless of the source of heat for extensional crustal melting, which may be multiple^66^, a first order consistency between modeled and observed magmatic products is the essential requirement for assessing a possible surface processes forcing. Later decompression melting of the asthenosphere begins prior to continental breakup and continues during syn- and post-breakup phases. Overall, the topography produced by the model is consistent with that of rift flanks and basins observed in natural case studies, where the rift basin is filled at the expenses of the eroded rift shoulders (Figs. 4 and 5).

**Figure 6:**
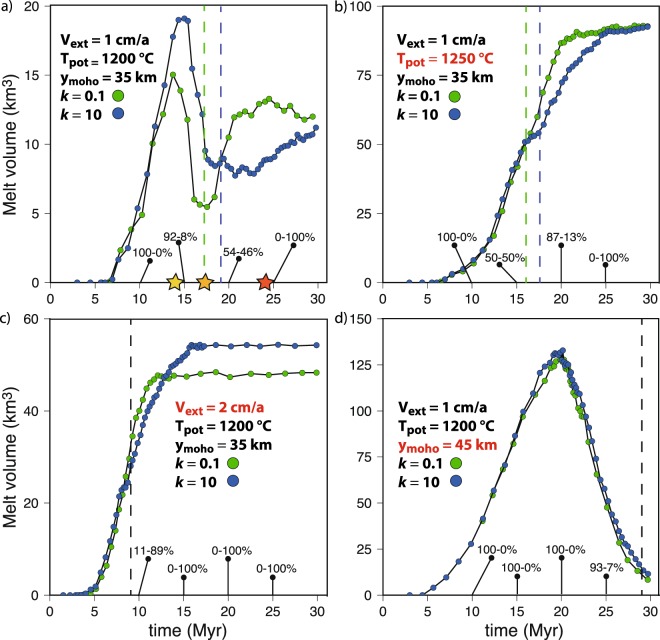
Parametric study results summary: (**a–d**) Melt volume (per unit distance in the direction perpendicular to those of the model domain) integrated across the model domain vs. time for eight selected numerical simulations. Imposed *V*_*ext*_, *T*_*pot*_, *y*_*moho*_, and *k* values for each numerical simulation are shown on the plots. Green and blue dot-lines denote numerical simulations with inefficient and efficient surface processes, respectively. The mean percentage of crustal and mantle melts at selected timesteps (10, 15, 20, and 25 Myr) is respectively displayed on each plot. The vertical dashed lines indicate the time of continental breakup (i.e. final rupturing of the extended continental mantle and crustal material). If black, then continental breakup in the simulation with efficient and inefficient surface processes occur at the same time. The yellow, orange and red stars in panel (**a**) (reference model setup) show the timing of panels (**a**), (**b**) and (**c**), respectively, in Fig. 7. The figure was generated with MATLAB version R2017 and assembled with Adobe Illustrator version 24.0.1.

**Figure 7:**
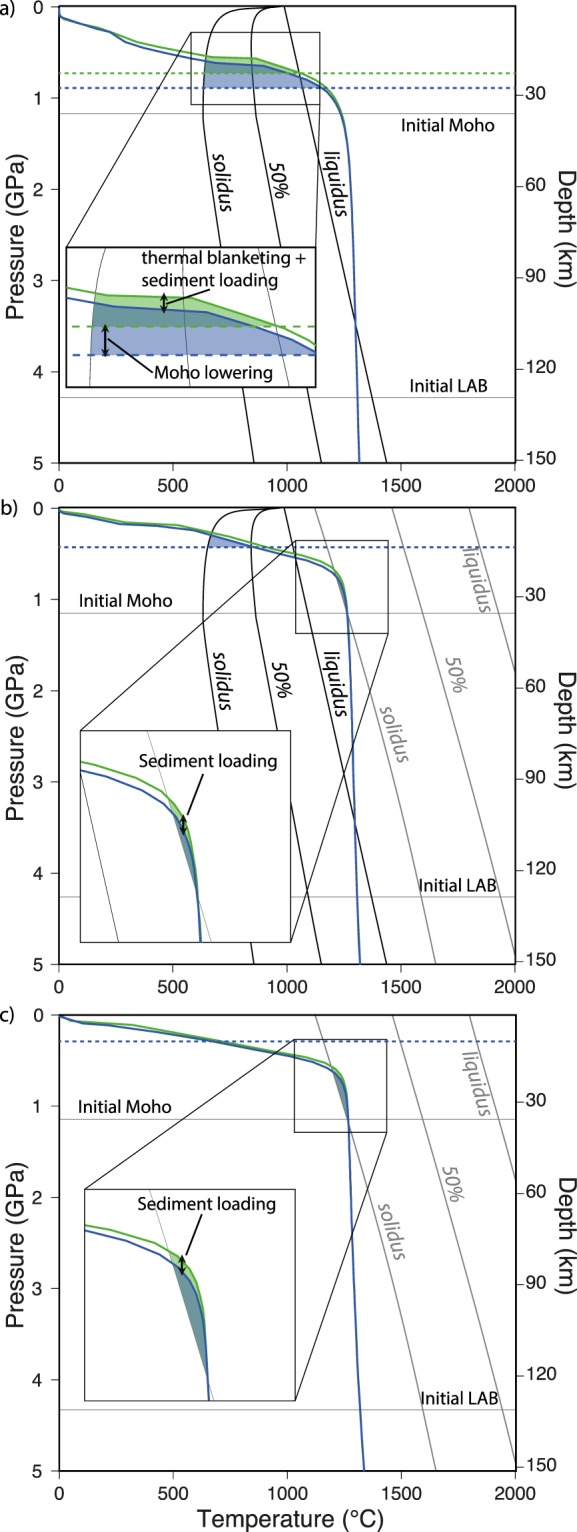
Melt generation: Reference model geotherm below the rift centre when efficient ($k=10$, blue line) and inefficient ($k=0.1$, green line) surface processes are accounted for and solidus-liquidus of crustal (black) and mantle (grey) material^64,66^. (**a**) Time equal to ~14 Myr (yellow star in the top-left panel of Fig. 6). (**b**) Time equal to ~17 Myr (orange star in the top-left panel of Fig. 6). (**c**) Time equal to ~24 Myr (red star in the top-left panel of Fig. 6). For clarity, mantle and crustal solidus and liquidus are not displayed in the top and bottom diagrams, respectively, because no mantle or crustal melting occur at those stages of the simulation. Horizontal dashed lines in panels (a) and (b) show the Moho depth. The figure was generated with MATLAB version R2017 and assembled with Adobe Illustrator version 24.0.1.

The results of the parametric study are summarized in Fig. 6. The estimated volume of melts generated during rifting at 1 cm/yr with the Moho at 35 km depth and mantle potential temperature of 1200 °C (i.e. reference model) is subject to strong variations depending on the redistribution of the surface masses (Fig. 6a). Early crustal melting at Moho levels is enhanced by efficient sediment delivery to the rift basin, which dampens crustal thinning, increases subsidence and facilitates basal heating due to asthenospheric upwelling through the thinning mantle lithosphere (Figs. 5a and 7a). In turn, a greater crustal thickness and higher Moho temperature due to efficient surface processes promote ductile straining of the crust with respect to brittle rupturing, leading to delayed continental breakup (Fig. 6a). During later stages of the model evolution, mantle melting is inhibited by efficient sediment delivery to the rift basin (Fig. 6a) because an increasing sedimentary load dampens decompression of the upwelling asthenosphere, thereby reducing the portion of the geotherm above the mantle solidus/liquidus (Fig. 7b,c).

When the reference model setup (Fig. 6a) is modified to account for a higher mantle potential temperature (e.g. 1250 °C, Fig. 6b), faster extensional velocity (e.g. 2 cm/a, Fig. 6c), and thicker crust (e.g. 45 km, Fig. 6d), the maximum amount of melts produced increases by ~ five-, three- and six-fold respectively. Enhanced partial melting of both the crust and asthenosphere when a higher mantle potential temperature with respect to the reference model is accounted for (Fig. 6b) is expected, because this implies a warmer geotherm across the model domain. Doubling the extensional velocity with respect to the reference model leads to faster lithospheric rupturing (breakup after only ~8 Myr compared to ~17 Myr in the reference model), which in turn enhances mantle melt production by faster upwelling and decompression rates (Fig. 6c). Increased crustal thickness with respect to the reference model implies crustal material to greater depths and higher temperatures and, thus, enhanced crustal melt production (Fig. 6d). Similar trends in the modulation by surface processes to the amount of melts produced are observed when the mantle potential temperature is 1250 °C, the extension velocities are 2 cm/yr or the initial crustal thickness is 45 km (Fig. 6b–d). However, these trends appear smoothened in the former two cases (Fig. 6b,c) and significantly dampened in the latter case (Fig. 6d), when crustal melting dominates on mantle decompression melting and particularly voluminous melts are produced.

## Discussion

**Figure 8:**
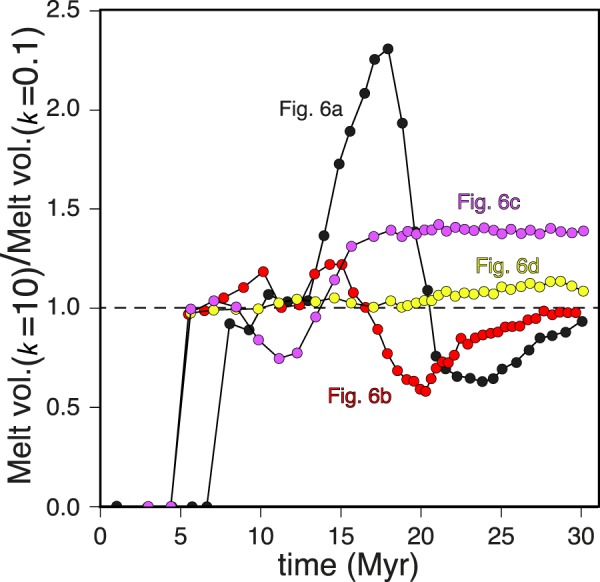
Surface processes forcing on extensional rock melting: Time evolution of the ratio between melt volume integrated across the model domain for the simulations in Fig. 6 when efficient ($k=10$) and inefficient ($k=0.1$) surface processes are accounted for. For the specific set of conditions imposed in the reference model (Fig. 6a), surface processes appear to more than double the amount of crustal melts and reduce the amount of mantle melts by ~50% (see text for more detail). The figure was generated with MATLAB version R2017 and assembled with Adobe Illustrator version 24.0.1.

Coupled thermo-mechanical and landscape evolution numerical modeling allows reproducing histories of continental rifting and partial melting consistent with those of previous studies and observations from natural settings^4,60,67–69^. It is thus possible to draw general relationships from the numerical results and evaluate their applicability to and implications for natural rifts. Without feedbacks between surface and subsurface processes, the topographic anomalies generated during continental rifting would be rapidly reset^4^. To a first approximation, the rift flanks and the underlying Moho rise by approximately the ~5/6^th^ of the crustal thickness lost by erosion within a given time window and, by the same principle, the crust bends downward as the rift basin is filled, which generates more accommodation space for sediments^4,70^. This study, however, shows that the feedbacks between surface and deep Earth processes in rifting settings are not limited to topographic features, but also involve partial rock melting (Figs. 4 and 6–8). If thermal blanketing due to efficient rift basin filling may rise lower crustal temperatures by ~50–100 °C (ref. ^4^), thereby contributing to enhance crustal partial melting, sediment loading in the rift basin lowers the geotherm with respect to the crustal solidus, which dampens or even overrides the effect of thermal blanketing (Fig. 7). The competing effects of thermal blanketing and solidus lowering on crustal melting, however, is outpaced by flexural bending of the Moho in a stretching and warming lithosphere, which supplies additional crustal material at near/above-solidus conditions, ultimately enhancing the amount of crustal melts (Figs. 6 and 7). Lithospheric stretching at rates of a few cm/yr forces the asthenosphere to upwell and decompress at a similar rate (Figs. 4 and 5). Decompression partial melting of the asthenosphere, however, is dampened by surface loading due to efficient filling of the rift basin (Fig. 6). The dampening is a function of the rate of basin deepening/filling, the sediment density, and the surface-to-depth stress change transfer of the rift system. For the reference model setup, the crustal melt production is roughly doubled and the mantle melt production is reduced by ~50% when surface processes redistribute the surface masses so efficiently to nearly reset the topography through time (i.e. the rate of erosion/deposition is similar that of tectonically-controlled surface uplift/subsidence) (Figs. 5, 6 and 8). This upper bound estimate may be used as a reference to infer possible modulations by surface processes to rock melting in natural rifting settings, which, for a given erosion/deposition rate, appears to be inversely correlated to the extensional velocity, mantle potential temperature and initial Moho depth (Fig. 6). Particularly noteworthy is that, while faulting and hence brittle-plastic deformation plays an important role in setting the rate and amount of melt percolation along fracture zones^22,71^, melt percolation reduces the brittle-plastic strength of rocks through an increase in pore fluid pressure^72^. This feedback, which further localises deformation along weakening fault zones and facilitates the ascent of partially molten rocks through the lithosphere, is not taken into account in the numerical models presented here, which has two main implications. First, the reference upper bound estimate regarding the possible modulation of partial melting by surface processes (Fig. 8) is likely underestimated to some extent. Second, thermal blanketing and flexural stresses due to abundant sediment supply to the rift basin weaken the lithosphere and favour prolonged viscous stretching upon abrupt brittle/plastic rupturing^4^, thereby explaining the retardation of continental breakup (Fig. 6). However, since brittle-plastic strain localisation due to melts-rocks interactions is not taken into account, the influence of surface processes on the timing of lithospheric rupturing cannot be properly assessed. I anticipate that future studies will investigate the relative contributions of thermal blanketing and melts-rocks interactions modulated by surface processes in affecting the timing of continental breakup in extensional settings.

In terms of driving mechanisms, the surface processes forcing on rock melting quantified here is similar to the proposed deglacial or eustatic control on extensional rock melting in, for instance, Iceland, the Mediterranean, or the Australian-Antarctic ridge^33,36–38^. However, the improvement with respect to previous studies is that the parametric investigation performed with fully coupled landscape evolution and thermo-mechanical geodynamic models allows constraining a range of conditions in which the surface processes forcing on extensional rock melting is likely conspicuous. That is, when the crust is thinner than ~40 km, the extension rate is slower than ~2 cm/yr and the mantle potential temperature is below ~1230 °C (Fig. 6). While the first two conditions are commonly observed^56,57^, the global mean mantle potential temperature at mid-oceanic ridges was estimated as ~ 1360 °C (refs. ^73–75^). However, long wavelength temperature variations in the sub-lithospheric convective upper mantle are at least $\pm$ ~ 200 °C (refs. ^76–78^). Therefore, plausible mantle potential temperatures range between ~ 1160–1560 °C (ref. ^13,45^). According to ref. ^76,78^ negative anomalies with respect to the mean value may reach up to ~ −250 °C and ~ −150 °C at “cold” mantle regions such as non-ridge domains or nearby subduction zones, respectively. Underneath the continents and far from mantle plumes, the mantle potential temperature is estimated as ~1200 °C, that is at least 100 °C cooler than that beneath the oceans^79^. The mantle geotherm below the Corinth Rift and nearby the Gulf of Lion rift system, for instance, may be cooler than the 1100 °C adiabat^80^, possibly due to the proximal Hellenic and Alpine-Apennine slabs. Estimated mantle temperatures for eastern North America and offshore of Iberia suggest mantle geotherms significantly below the 1280 °C mantle adiabat prior and during rifting, which is consistent with the magma-poor structure of the Iberia-Newfoundland margin^79,80^ ref. ^81^ estimate the modern average geotherm for the western United States as asymptotic to the 1300 °C adiabat due to the influx of oceanic asthenosphere from the Gulf of California. This scenario implies that the mantle potential temperature at the base of the lithosphere prior to oceanization was closer to 1200 °C than to 1300 °C^79^. These lines of evidence suggest syn-extensional mantle potential temperatures as low as ~ 1230 °C, or even cooler, in the Mediterranean region, the Iberia-Newfoundland margin and the Gulf of California. I propose that in these and possibly other extensional settings such as the South China Sea, surface processes may have affected partial melting by up to doubling the amount of melts produced (Figs. 6a and 8)^82^. I further speculate that the mass redistribution due to surface processes across these settings may have contributed to the rift geometry, migration, segmentation or failure, for instance through modulation of crustal or mantle melting and associated magma-assisted lithospheric rupturing^19,72,83^ or the modulation of inherited structures^84,85^. If correct, an important implication is that the role of climate-controlled surface processes in conditioning the surface expressions of plate tectonics are not limited to the modulation of the topography^27,86^, but also extend to continental drifting and, thus, the current global plate configuration.

Recent investigations show a correlation between the worldwide rifts or mid oceanic ridges length over the last ~200 Ma and atmospheric CO_2_ proxies^42,43^, in turn suggesting that extensional rock melting contributes to setting long term global climate trends. However, climate is the main driver of surface processes. Thus, the surface processes forcing on extensional rock melting investigated here may involve a previously unknown feedback mechanism between the Solid Earth and the Surface Earth, which likely conditioned the long term (multi-Myr) evolution of the Earth system. A decrease in solidus temperatures of peridotites in presence of CO_2_^87–89^ would enhance this newly-recognized surface processes feedback on climate via extensional melting. Since the magma migration velocities and signal propagation rates in sedimentary systems commonly reach up to the tens of cm per year^51,90^, it is likely that the long term surface processes forcing on extensional rock melting addressed here also finds expression at shorter (e.g. centennial to multi-millenial) timescales. Milankovitch frequencies in tephra or bathymetric records worldwide^37,91^ corroborate this speculation. However, in order to validate these assertions, more advanced erosion-deposition physical laws including, for instance, fluvial and dispersed sediment transport, respectively dominant in channels and hillslopes^92^, are required. Accounting for variable rock erodibility, precipitation rates and river network geometries in space and time^93^ would further improve the description of the modifications of the surface load, in turn allowing to better constrain the surface processes forcing on extensional rock melting. Because unravelling the interactions between plate tectonics and climate through the geological carbon cycle is undoubtedly one of the major challenges in the Earth sciences^44^, future works that will address the many possible nuances in the first order relationships between surface processes and extensional rock melting identified here are warranted.

## Conclusions

The numerical models presented here show that the surface mass redistribution by erosion and sediment deposition conditions rock melting across extensional settings. Prominent surface processes may double the amount of magma generated by crustal melting and reduce by ~50% the amount of magma generated by mantle decompression melting, if continental stretching is slower than ~2 cm/yr, the crust is thinner than ~40 km, and the mantle potential temperature is below ~1230 °C. The limitations the numerical model is subject to, particularly those regarding the influence of fluids on the plastic deformation of rocks, are such that these inferences may be underestimated and that the range of conditions in which surface processes significantly affect extensional rock melting may be wider. The implications of these findings are, at least, twofold. First, climate-controlled erosion, sediment transport and deposition, condition the structural evolution of extensional settings not only through effects on the topography and subsurface stress and strain field^4^, but also through effects on partial rock melting and associated magmatic and rheological changes. Second, a previously unrecognized mutual feedback between climate and tectonics exists and takes place through a control of surface processes on extensional rock melting and of rift magmatism on surface processes via climatic effects.

## Data Availability

No datasets were generated or analysed during the current study, but the codes used are available from the corresponding author on request.
